# NanoLuc Binary Technology as a methodological approach: an important new tool for studying the localization of androgen receptor and androgen receptor splice variant V7 homo and heterodimers

**DOI:** 10.1186/s12885-024-12110-2

**Published:** 2024-03-19

**Authors:** Juan Guzman, Katrin Weigelt, Angela Neumann, Philipp Tripal, Benjamin Schmid, Zoltán Winter, Ralph Palmisano, Zoran Culig, Marcus V. Cronauer, Paul Muschler, Bernd Wullich, Helge Taubert, Sven Wach

**Affiliations:** 1 Department of Urology and Pediatric Urology, Uniklinikum Erlangen, Friedrich-Alexander-Universität Erlangen-Nürnberg, Erlangen, 91054 Germany; 2 Comprehensive Cancer Center Erlangen-EMN (CCC ER-EMN), Erlangen, 91054 Germany; 3 Optical Imaging Centre Erlangen, Friedrich-Alexander-Universität Erlangen-Nürnberg, Erlangen, 91054 Germany; 4 Department of Urology, Division of Experimental Urology, Medical University of Innsbruck, Innsbruck, 6020 Austria; 5 Institute of Pathology, Universitätsklinikum Bonn, Universität Bonn, Bonn, 53127 Germany; 6 Promega GmbH, Walldorf, 69190 Germany

**Keywords:** Androgen receptor, Androgen receptor variant 7, Prostate cancer, NanoLuc Binary Technology, Homodimer, Heterodimer

## Abstract

**Background:**

The androgen/androgen receptor (AR)-signaling axis plays a central role in prostate cancer (PCa). Upon androgen-binding the AR dimerizes with another AR, and translocates into the nucleus where the AR-dimer activates/inactivates androgen-dependent genes. Consequently, treatments for PCa are commonly based on androgen deprivation therapy (ADT). The clinical benefits of ADT are only transitory and most tumors develop mechanisms allowing the AR to bypass its need for physiological levels of circulating androgens. Clinical failure of ADT is often characterized by the synthesis of a constitutively active AR splice variant, termed AR-V7. AR-V7 mRNA expression is considered as a resistance mechanism following ADT. AR-V7 no longer needs androgenic stimuli for nuclear entry and/or dimerization.

**Methods:**

Our goal was to mechanistically decipher the interaction between full-length AR (AR-FL) and AR-V7 in AR-null HEK-293 cells using the NanoLuc Binary Technology under androgen stimulation and deprivation conditions.

**Results:**

Our data point toward a hypothesis that AR-FL/AR-FL homodimers form in the cytoplasm, whereas AR-V7/AR-V7 homodimers localize in the nucleus. However, after androgen stimulation, all the AR-FL/AR-FL, AR-FL/AR-V7 and AR-V7/AR-V7 dimers were localized in the nucleus.

**Conclusions:**

We showed that AR-FL and AR-V7 form heterodimers that localize to the nucleus, whereas AR-V7/AR-V7 dimers were found to localize in the absence of androgens in the nucleus.

**Supplementary Information:**

The online version contains supplementary material available at 10.1186/s12885-024-12110-2.

## Background

The AR gene is located on chromosome Xq11-12 and is organized into 8 canonical exons, that encode the AR protein, a ligand inducible transcription factor of the steroid receptor superfamily. Like all steroid receptors, the AR has a modular structure composed of 4 distinct functional domains: an amino-terminal or transactivation domain (NTD/TAD, encoded by exon 1) and a central DNA binding domain (DBD, exons 2–3), which carry two zinc finger motifs involved in DNA-recognition and receptor dimerization, as well as a carboxy-terminal ligand binding domain LBD (predominantly exons 5–8). The NTD/DBD-core and LBD are interconnected by a small flexible linker, the hinge region (HR, exon 4). The latter harbors the carboxy-terminal end of a lysine/arginine rich bipartite nuclear localization signal (NLS) spanning between the DBD (exon 3) and the HR (exon 4) [1].

In the absence of a ligand, AR is bound to a heat shock protein (HSP) complex, which retains the AR-protein in an inactive conformation in the cytoplasm. Upon androgen/ligand-binding the AR dissociates from parts of the HSP-complex and converts to an active form where the NTD interacts with the C-terminal LBD. The intramolecular N/C-interaction is followed by a rapid nuclear translocation [2–5]. In the canonical AR-signaling pathway, AR-proteins are presumed to dimerize in the nucleus. AR-dimers bind to androgen response elements (AREs) in the cis-regulatory regions of androgen-dependent genes. The full AR-transcriptional complex is completed by the recruitment of coregulators, which ultimately results in the regulation of target gene transcription [6–9].

Early functional in vitro studies have shown a high constitutive transcriptional activity in several AR-constructs in which the LBD has been artificially deleted [10, 11].

Over the past decade, various mRNAs from C-terminally truncated, constitutively active AR-variants have been identified in cell lines, patient xenografts and primary prostate cancer tissue specimens [12–14]. In prostate cancer, the generation of C-terminally truncated AR-variants (AR-Vs) is predominantly driven by the splicing of cryptic exons [15], exon skipping [16] or genetic rearrangements [17]. Silencing of members of the splicing machinery results in a decrease in the expression levels of key oncogenic splice variants (e.g. AR-V7) and dysregulation of the splicing machinery is associated with the aggressiveness of PCa [18].

To date, clinical interest in the role of constitutively active AR splice variants such as AR-V7 or AR-v567es has grown rapidly. Devoid of a functional LBD, these AR-Vs are unable to bind and respond to androgens or antiandrogens. Consequently, conventional endocrine therapies targeting androgen synthesis and/or androgen binding are prone to failure once these AR-Vs are overexpressed in prostate cancer [12, 19–21]. Unfortunately, the molecular mechanisms by which AR-Vs are regulated in CRPC are not fully understood [22, 23].

The best characterized constitutively active AR-splice variant is AR-V7, also termed AR3. Initially, discovered in the castration resistant prostate cancer cell line 22Rv1. AR-V7 is expressed at low levels in primary PCa but is increased in CRPC [24, 25]. Enzalutamide targets the AR LBD and thus is not expected to affect AR-V7 [19]. Taken together, these findings suggest that AR-V7 confers resistance to second generation endocrine treatments such as abiraterone or enzalutamide. The AR-V7 splice variant comprises canonical exons 1–3 and expresses a cryptic exon 3 (CE3) [12]. The CE3 of AR-V7 encodes a 16 amino acid peptide that replaces the HR and LBD, encoded by canonical exons 4–8 of the AR. Most AR-Vs no longer express exon 4, which includes the carboxyterminal end (^630^RKLKK^634^) of the bipartite nuclear localization signal (NLS) [26]. Consequently, AR-Vs that do not express exon 4 are expected to be located predominantly in the cytoplasm.

Surprisingly, AR-V7 constitutively localizes to the nucleus and has transcriptional activity devoid of a functional NLS [22]. As suggested by Chan et al., AR-V7 displays enhanced nuclear localization because the amino acids Lys-629 and Arg-631 of the CE3 that are able to reconstitute the carboxyterminal region of the bipartite AR-NLS [27]. Moreover, Chan and colleagues reported that truncated AR-Vs, expressing an intact AR NTD/DBD-core (exon 1–3) exhibit a basal level of nuclear localization, sufficient for androgen-independent transcriptional activity. There is experimental evidence, that AR-V7 can also form heterodimers with transcription factors such as ZFX, whose nuclear localization sequences could further enhance the nuclear entry of AR-V7 [28].

Recently, Cao et al. studied the formation of AR-FL-and AR-V7- homodimers and AR-FL/AR-V7 heterodimers in AR-negative COS-7 cells [29]. In the absence of androgens, AR-V7 facilitates the nuclear translocation of AR-FL. In addition, enzalutamide, a second-generation anti-androgen, inhibited AR-FL translocation to the nucleus, but this effect was mitigated in the presence of AR-V7 [19]. Furthermore, the nuclear localization of AR-V7/AR-V7 was not affected by androgens or enzalutamide [29].

The stepwise dimerization of AR-FL has been described in detail by van Royen et al. [2]. In addition, Xu and coworkers analyzed the ability of AR-V7, AR-v567es and AR-FL to form homo-/heterodimers in the absence of androgens in PC3 cells [4]. Their study indicated that AR-V/AR-FL dimerization is mediated by both a DBD/DBD and an N/C (NTD-LBD) interaction. However, since AR-Vs have lost their LBD, the LBD is provided by AR-FL and the NTD from AR-V in the heterodimer [4]. AR-V7/AR-V7 homodimers exhibit only DBD-DBD interactions. AR-V7/AR-FL heterodimers and AR-V7/AR-V7 homodimers were primarily detected in the nuclei of PC-3 cells. AR-FL/AR-FL homodimers were observed only after DHT treatment and mostly occurred in the nuclei of PC-3 cells.

Özgün et al. investigated the DNA binding of AR-FL, AR-V7 homodimers and AR-V7/AR-FL heterodimers. AR-FL/AR-V7 heterodimers readily form in the nucleus via intermolecular N/C (NTD-LBD) interactions. However, DNA binding occupancy is determined by protein monomers, not homodimers or heterodimer complexes [30].

However, at what time-point AR-FL/AR-FL homodimers enter the nucleus following DHT treatment is unclear. Furthermore, we do not know whether AR-V7/AR-FL heterodimers form in the cytoplasm or if they enter the nucleus as AR-V7/AR-V7 and AR-FL/AR-FL homodimers and perform a partner swap in the nucleus. We applied NanoLuc Binary Technology (NanoBiT) for highly sensitive intracellular detection of protein:protein interactions [31] for AR-FL/AR-FL and AR-V7/AR-V7 homodimers and AR-FL/AR-V7 heterodimers. AR-FL/AR-FL homodimers formed in the cytoplasm. AR-FL/AR-FL homodimers translocated into the nucleus within 15 min after DHT treatment. AR-V7/AR-V7 homodimers were constitutively located in the nucleus, and neither DHT nor enzalutamide affected the localization of AR-V7/AR-V7 or its status as a dimer. However, our data indicate that AR-V7/AR-FL heterodimers form in the nucleus after AR-FL homodimers are translocated to the nucleus.

## Methods

### Cloning of tagged androgen receptor constructs

To study the interaction between the androgen receptor and splice variant AR-V7 homo or heterodimers, we used the NanoBiT PPI MCS Starter System (Promega, Madison, WI, USA). Primers were designed to amplify the coding regions of AR-FL and AR-V7 via PCR and to add specific restriction sites. After agarose gel purification, the PCR fragments and target vectors were restriction digested to allow in-frame insertion of the coding regions into expression vectors containing large BiT (LgBiT) and small BiT (SmBiT) fragments (Suppl. Fig. S1). The sequences of the amplification primers are listed in Table 1. All the expression vectors were verified by sequencing. A list of the plasmids used and cloned in this study is provided in Suppl. Table S1. The HSV-TK promoter drives the expression of the fusion proteins (Suppl. Figs. S2 and S3). The SmBiT-PRKACA:LgBiT-PRKAR2A pair served as a positive control, in which fusion partners interact without adding a compound. PRKACA is the catalytic subunit α of protein kinase A, and PRKARS2A is the cAMP-dependent protein kinase type II-alpha regulatory subunit.

**Table 1:** PCR primers

Cloning Primer	MCS Restriction site	Sequence
N-Terminal-AR/ARV7-FW	Xhol	5’-CTCGAGATGGAAGTGCAGTTAGGGCTGG-3’
N-Terminal-AR-RV	Bglll	5’-AGATCTGCTTCACTGGGTGTGGAAATAGATGG-3’
N-Terminal-AR-V7-RV	Bglll	5’-AGATCTTCTTCAGGGTCTGGTCATTTTGAGAT-3’
C-Terminal-AR/ARV7-FW	Bglll	5’-AGATCTATGGAAGTGCAGTTAGGGCTGG-3’
C-Terminal-AR-RV	Xhol	5’-CTCGAGCCCTGGGTGTGGAAATAGATGGGCTTG-3’
C-Terminal-AR-V7-RV	Xhol	5’CTCGAGCCGGGTCTGGTCATTTTGAGATGCTTGCA-3’

All primers used were obtained from Biomers.net (Ulm, Germany). FW is the forward primer and RV the reverse primer

### Cell culture conditions HEK-293

HEK-293 cells were cultured in DMEM (Sigma‒Aldrich, Darmstadt, Germany) supplemented with 10% charcoal-stripped fetal bovine serum (Sigma‒Aldrich), 1% penicillin‒streptomycin and 20 mM HEPES (Pan Biontech, Aidenbach, Germany). HEK-293 cells were kindly provided by Dr. Zoran Culig/ Medical University of Innsbruck. Cells were regularly tested for mycoplasma infection.

For expression of tagged AR-FL and AR-V7 constructs, 1 µg (0.5 µg of each LgBiT and SmBiT interaction partner) was transfected into subconfluent grown HEK-293 cells using the jetPRIME transfection system according to the manufacturer’s recommendations (Polyplus Transfection, Illkirch, France). Twenty-four hours after transfection, the cell culture medium was replaced, and the cells were stimulated with 1 nM DHT or 10 µM enzalutamide for another 24 h. As a control each of the four LgBiT fusion constructs was coexpressed with HaloTag-SmBiT. Since all four control pairs displayed very similar signals, we continued our experiments with one representative negative control.

### Luminescence in plates

Luminescence measurements were carried out with a TECAN Infinite M200 Pro (Tecan, Männedorf, Switzerland) plate luminometer. Briefly, HEK-293 cells were seeded in white plates at a density of 10,000 cells/well, incubated overnight and transfected with AR expression constructs or control constructs. After 24 h, Nano-Glo Live Cell Substrate (Promega) was added, and protein‒protein interactions were induced by adding DHT (1 nM final concentration). An intact NanoLuc luciferase protein tag was generated through direct protein‒protein interaction. Luminescence was measured in the luminometer, preheated to 37 °C, over a period of 2 s and normalized as counts per second (CPS).

### Confocal microscopy

We used confocal microscopy to assess the subcellular localization of tagged AR proteins and the localization shift following stimulation with DHT. For this purpose, HEK-293 cells were seeded on coverslips coated with poly-L-lysine and placed in 6-well plates. HEK-293 cells were cotransfected with different androgen receptor full-length (AR-FL) and androgen receptor splice variant-7 (AR-V7)-tagged constructs (Suppl. Table 1). Twenty-four hours after transfection, the cells were stimulated with 1 nM DHT for 0, 15, 30 or 60 min. Immediately after stimulation, the cells were fixed with 4% paraformaldehyde (PFA) for 20 min at room temperature and washed three times for 5 min each with phosphate-buffered saline (PBS). Then, the cells were permeabilized with 0.2% Triton X-100 solution for 10 min at room temperature. The samples were blocked with 1% BSA + 0.1% Triton X-100 solution for 20 min at room temperature. To detect the tagged AR protein, we used a NanoLuc Luciferase Antibody (R&D Systems, Clone 965,853; 1:500), which detects both the LgBiT component and the complemented NanoLuc, overnight at 4 °C.

After overnight incubation, the samples were washed with PBS and incubated with the secondary Alexa Fluor 488 conjugated anti-mouse IgG antibody (Invitrogen, Darmstadt, Germany; A-11001; 1:1000) diluted in blocking solution (1% BSA + 0.1% Triton X-100) for 60 min at room temperature. Again, the samples were washed with PBS and preserved in one single step by using mounting medium (Carl Roth, Karlsruhe, Germany) supplemented with DAPI (4',6-diamidino-2-phenylindole) to stain cell nuclei. The samples were analyzed via fluorescence microscopy with a Leica SP5 II (Leica, Wetzlar, Germany). Suppl. Table 2 shows an overview of the timeline from seeding to mounting of the HEK-293 cell line.

### Live cell luminescent imaging

For live cell luminescence imaging, HEK-293 cells were plated on chambered coverslips coated with poly-L-lysine as described previously. After 24 h of incubation, the cells were cotransfected with the AR-FL and AR-V7 NanoBiT constructs (Suppl. Table 1) and incubated overnight. Immediately before imaging, the cells were stimulated with 1 nM DHT and NanoGlo live-cell substrate (Promega). Luminescence was recorded and integrated for a total of 90 min with an interval of 10 min and an exposure time of 10 s using a Leica DMi8 TIRF Widefield Fluorescence Microscope (Leica) equipped with an EMCCD camera (Andor, Oxford Instruments, iXon Ultra, Abingdon, UK).

## Results

### Characterization of AR-FL and AR-V7 via the NanoBiT protein‒protein interaction assay

**Fig. 1:**
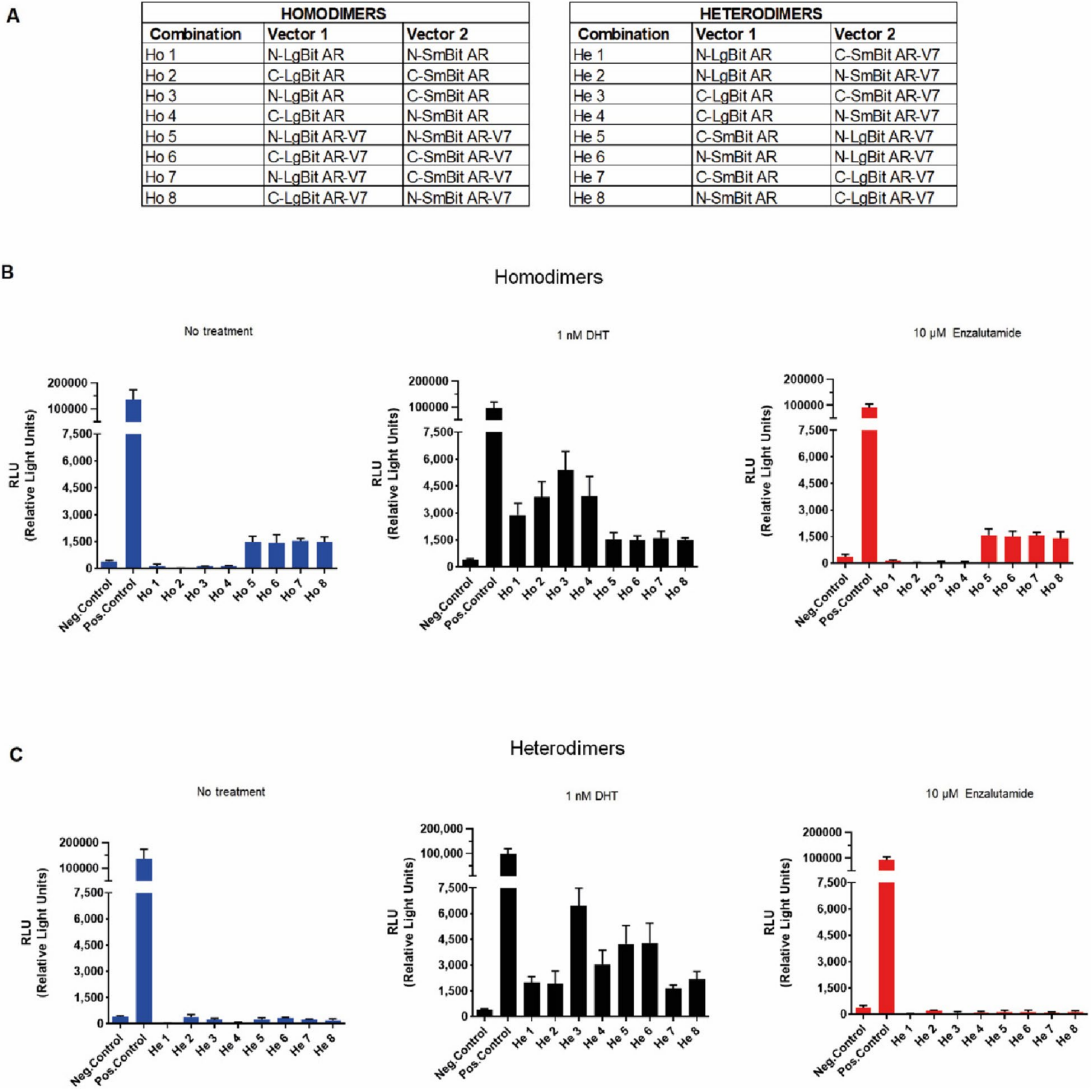
Nano**-**Glo® Live Cell assay.** A** HEK**-**293 cells were transfected with all possible combinations of homo**-** and heterodimers (Ho and He) encoding the LargeBiT (LgBiT) and SmallBiT (SmBiT) subunits in the N- and C- terminal regions of the androgen receptor full length (AR) and androgen receptor splice variant 7 (AR**-**V7) proteins. **B-C** Luminescence measurements in HEK**-**293 cells transfected with AR**-**FL and AR**-**V7 homo and-heterodimers after treatment with 1 nM DHT or 10 µM enzalutamide. Luminescence in nonstimulated HEK**-**293 cells was set as base line. The data represent the mean ± SEM (*n* = 3)

Because the orientation of the NanoLuc Binary protein tag components influences the results of live cell luminescence reactions, we first generated AR**-**FL**-** and AR**-**V7**-**tagged protein expression constructs to include all possible combinations of homodimers and heterodimers. There were four different combinations of the AR**-**FL and AR**-**V7 homodimers (Ho 1–8) and eight different AR**-**FL/AR**-**V7 heterodimer combinations (He 1–8) (Suppl. Fig. S1, Fig. 1A). HEK**-**293 cells were transiently transfected with 1 µg of plasmid combinations (0.5 µg of each plasmid construct). Twenty-four hours after transfection, the cell culture medium was replaced, and the cells were stimulated with 1 nM DHT or 10 µM enzalutamide for another 24 h. Control cells were transfected with a negative control vector encoding HaloTag**-**SmBiT, a structurally stable protein that is expressed throughout the cell and coexpressed with the respective LgBiT fusion construct.

Without stimulation, the HEK-293 cells expressing tagged AR-FL (Ho 1–4) did not show any increase in luminescence signal compared to that of the negative control, confirming the lack of AR-FL dimerization in the absence of androgens. However, cells expressing tagged AR-V7 (Ho 5–8) displayed constitutive luminescence activity, which was elevated by 3.7**-**, 3.5**-**, 3.8**-** and 3.7**-**fold compared to that of the negative control. When the cells were stimulated with 1 nM DHT, all the AR**-**FL combinations displayed marked increases in luminescence 7.4**-**, 10**-**, 13.8**-** and tenfold -, compared to that of the negative control cells, whereas AR**-**V7 combinations showed 3.9-, 3.8-, 4.0- and 3.8-fold luminescence that was identical to that of the control cells. This finding again confirmed that AR-FL dimers form only in the presence of androgens, whereas the extent of AR**-**V7 dimerization cannot be further enhanced.

Conversely, anti-androgen treatment with 10 µM enzalutamide, did not affect the dimerization of AR-FL monomers or inhibit the dimer formation of AR-V7, as these constructs consistently produced 4.3**-**, 4.1**-**, 4.2**-** and 3.8**-**fold greater luminescence than that of negative control**-**transfected cells (Fig. 1B).

Next, we tested the ability of AR**-**FL/AR**-**V7 heterodimers to form when the binary NanoLuc components were split between AR**-**FL and AR**-**V7. Without stimulation, none of the eight distinct combinations produced any increase in the luminescent signal compared to that of the negative control cells. This finding demonstrated that, in the absence of androgens, no heterodimer was formed. After stimulation with 1 nM DHT, all 8 combinations displayed increases in luminescence of 5**-**, 4.9**-**, 16.5**-**, 7.8**-**, 10.8**-**, 11**-**, 4.2**-** and 5.5**-**fold compared to that of the control cells (Fig. 1C).

In a treatment combining DHT and enzalutamide the dimerization of the AR**-**FL could be efficiently inhibited, while in accordance with previous experiments AR**-**V7 dimerization status remained unaffected. Interestingly, the heterodimer AR**-**FL/AR**-**V7 formation was also impaired but to a lesser extent than as detected in the AR**-**FL homodimer (Suppl. Fig. S4).

In summary, we confirmed that AR**-**FL resides as a monomer in the cytoplasm in the absence of androgens and that DHT readily stimulates dimer formation. AR**-**V7 forms a homodimer in the absence of androgen, and this dimer formation can neither be further stimulated by androgens nor inhibited by enzalutamide. When both AR**-**FL and AR**-**V7 were present, no trace of heterodimer formation was observed in the absence of androgens.

For all further experiments, we selected the homodimer 3 (N**-**LgBiT AR/C**-**SmBiT AR), homodimer 5 (N**-**LgBiT AR-V7/N**-**SmBiT AR**-**V7) and heterodimer 3 (C**-**LgBiT AR/C**-**SmBiT AR**-**V7) plasmid combinations that exhibited the most pronounced increase in luminescence after stimulation of the protein‒protein interaction (Fig. 1B and C).

### Localization of recombinant AR and AR-V7 proteins

We subsequently investigated the subcellular localization of the AR**-**FL and AR**-**V7 proteins upon DHT stimulation. For this purpose, we used immunofluorescence (IF) and confocal microscopy. For detection purposes, we used an antibody specific for the NanoLuc luciferase protein. Importantly, the detection antibody binds to both the complemented NanoLuc luciferase and the LargeBiT fragment but not to the SmBiT fragment, so the subcellular localization information does not provide any information about possible dimer formation.

**Fig. 2:**
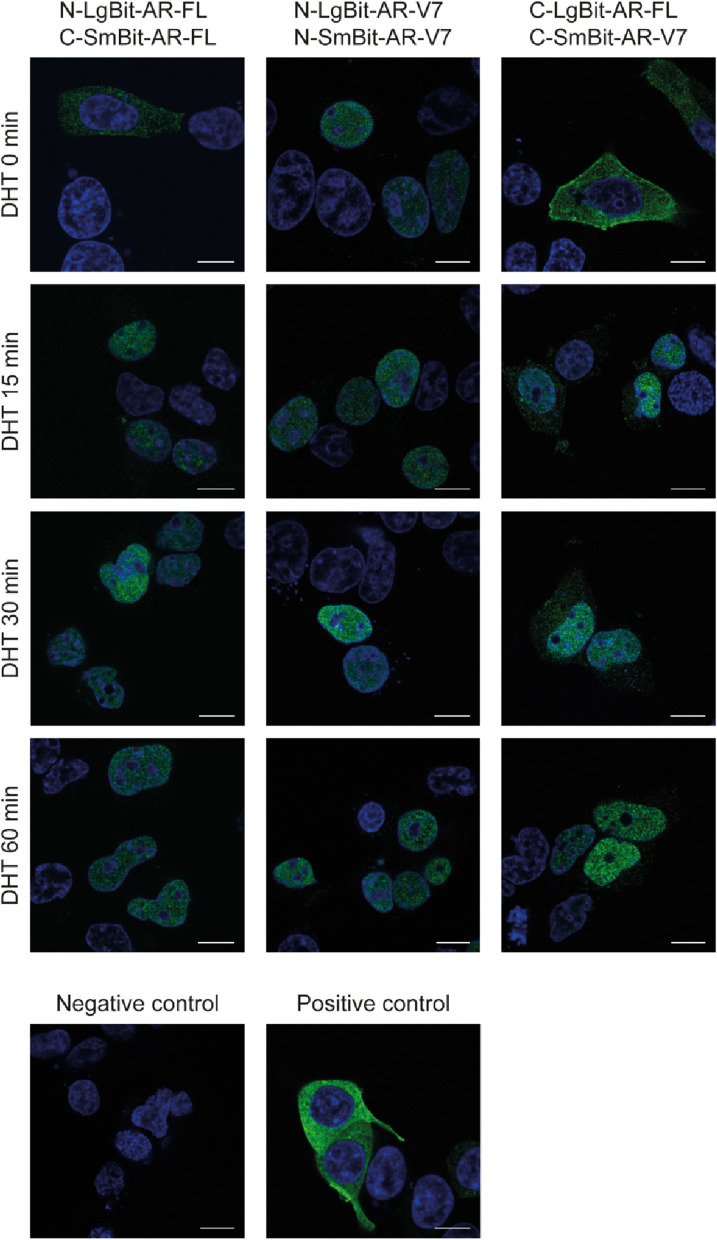
Immunofluorescence staining of NanoLuc luciferase. HEK-293 cells were transfected with the indicated combinations of plasmid vectors. Immunofluorescence (IF) staining was performed using NanoLuc and Alexa Fluor 488 antibodies. IF staining showing the subcellular localization of the AR-FL and AR-V7 proteins before and after androgen stimulation. The indicated combination constructs were transfected into HEK-293 cells, and IF staining was conducted 48 h after transfection. The cells were stimulated with 1 nM DHT for 0, 15, 30 or 60 min. DAPI was used for nuclear staining. The scale bar represents 10 µm. Only merged pictures are shown

Without androgen stimulation, we detected AR**-**FL monomers exclusively in the cytoplasm, whereas AR**-**V7 homodimers were exclusively located in the nucleus (Fig. 2). Upon stimulation with DHT, we observed the translocation of AR**-**FL from the cytoplasm to the nucleus. This translocation was almost complete in all cells after 15 min, and only small residues of AR**-**FL were detectable in the cytoplasm. This translocation was stable over the observed time period of 60 min. However, although this technique is suitable for tracking the subcellular location of tagged AR constructs, it cannot pinpoint the exact location where AR**-**FL dimerization takes place.

Regardless of whether AR-FL enters the nucleus as a monomer or as a dimer after DHT stimulation, we detected the presence of AR-FL/AR-V7 heterodimers. Considering that AR-V7 is located exclusively in the nucleus and that this localization is independent of any stimulation with DHT, these results indicate that the formation of heterodimers occurs in the nucleus after AR-FL monomers or homodimers are translocated into the nucleus (Fig. 2).

In summary, before DHT treatment, AR-FL is initially located in the cytoplasm, whereas AR-V7 is located in the nucleus. Upon androgen stimulation, AR-FL translocates into the nucleus, where it interacts with AR-V7.

### Bioluminescence imaging

**Fig. 3:**
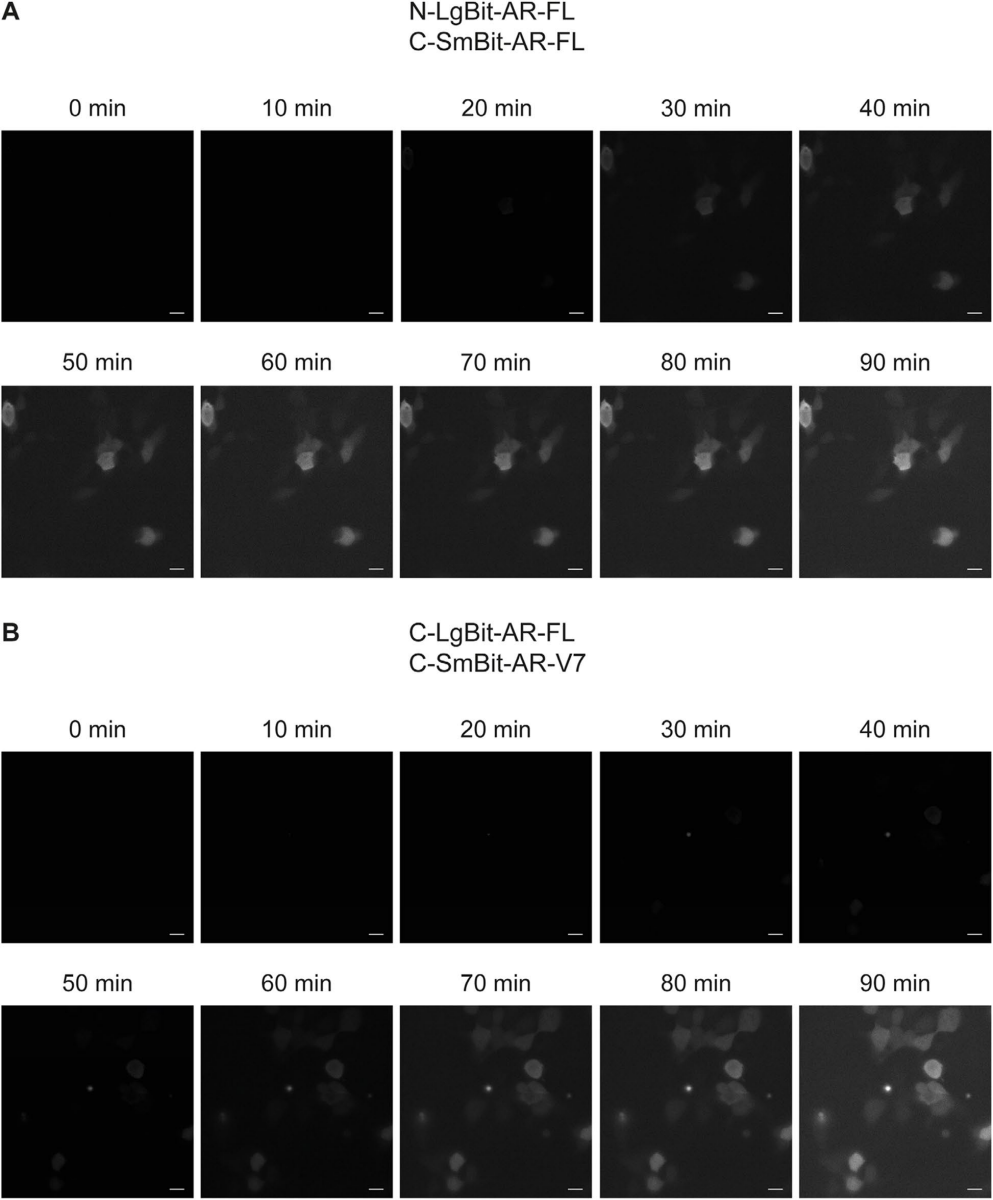
Bioluminescence imaging.** A-B** HEK-293 cells were transfected with homodimer 3 and heterodimer 3 plasmid combinations. Representative images of time course changes in luminescence every 10 min for 90 min after 1 nM DHT stimulation. The scale bar represents 10 µm

After 30 min of androgen stimulation, the integrated luminescence signal for the AR-FL homodimers crossed the detection threshold and was clearly detectable. AR-FL/AR-V7 dimers were detectable 50 min after stimulation. For both construct combinations, time course imaging allowed us to observe changes in luminescence intensity, but it was not possible to visualize the translocation of the luminescent signals (Fig. 3A-B). Unfortunately, with this method, we reached the technical limit of the visual detection threshold.

### Kinetics

**Fig. 4:**
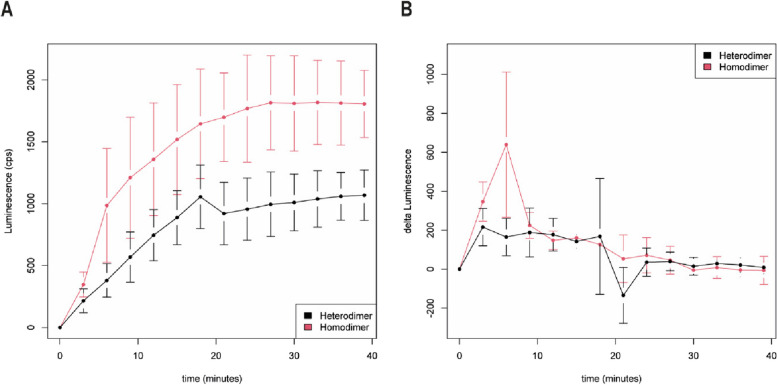
AR-FL homodimers and AR-FL/AR-V7 heterodimers form after DHT stimulation. **A** Luminescence measurements of AR-FL/AR-FL (red) and AR-FL/AR-V7 (black); **B** Kinetics of luminescence development. Changes in luminescence are displayed in a sliding 3 min window (mean ± SD)

We recorded the luminescence kinetics using the AR-FL homodimer and AR-FL/AR-V7 heterodimer formation. Immediately after androgen stimulation, luminescence was measured in 3 min intervals with a TECAN Infinite M200 PRO (Fig. 4). Therefore, the AR-FL homodimer showed a fast-detectable luminescence signal after 3–6 min, which continuously increased until 24 min after androgen stimulation, reached saturation and remained stable for the following measured time points.

A different picture was obtained for the AR-FL/AR-V7 heterodimer. Luminescence, as a marker for dimerization, increased more slowly in a linear fashion until 15 min and then remained at a constant level. However, the level of luminescence was lower for the AR-FL/AR-V7 heterodimer than for the AR-FL homodimer.

We suggest that interaction partners interact faster when they are in close proximity, as observed for AR-FL. However, when the interaction partners can only interact after translocation to the nucleus, as for AR-FL and AR-V7, they interact more slowly. The difference in the kinetics of the formation of AR-FL homodimers and AR-FL/AR-V7 heterodimers after DHT stimulation was detected directly (Fig. 4A), as was the difference in the luminescence (change in luminescence per 3 min interval) (Fig. 4B).

## Discussion

Based on the very strict dependency of PCa on AR signaling, most systemic therapies directly target the androgen receptor, androgen biosynthesis and/or interaction with androgens. As a consequence, tumors develop resistance to AR-targeted therapies. These resistance mechanisms can include AR overexpression, AR gene amplification, mutations in the ligand binding site of the AR, intracrine androgen synthesis and expression of constitutive splice variants [12, 32]. In particular, alternative splicing of AR is a specific mechanism that has attracted increased amounts of attention, as it is relevant to the progression of CRPC [12, 33]. The most prominent AR splice variant, AR-V7, has been described to mediate resistance to the second-generation anti-androgens enzalutamide and abiraterone [19]. However, nuclear AR-V7 expression can be detected in primary prostate cancer prior to long-term androgen deprivation and castration resistance [34]. A meta-analysis recently showed that the proportion of AR-V7-positive patients was significantly greater in CRPC patients than in newly diagnosed prostate cancer patients [35]. In addition, especially for hormone-sensitive PCa patients, the AR-V7-positive patients had a worse prognosis after first-line hormonal therapy and prostatectomy, as shown by shorter PFS and OS [35]. The expression of AR-V7 was associated with a poor prognosis and is an independent risk factor for reduced overall survival in mCRPC patients treated with endocrine therapy [36].

In our study, we focused on AR and AR-V7. In particular, their homodimerization and heterodimerization kinetics are important. We studied these interactions by using NanoLuc Binary Technology (NanoBiT), which can characterize protein–protein interactions in live cells, allowing real-time detection of complex formation [31].

We confirmed that AR-V7 homodimerization occurs in the absence of androgen and that its interaction cannot be further stimulated (dihydrotestosterone/DHT) or inhibited by an anti-androgen (enzalutamide). In contrast to the findings of other reports, describing AR-FL/AR-V7 dimerization in PC3 cells that do not require androgen stimulation [4]), we observed that the formation of AR-FL/AR-V7 heterodimers strictly occurs upon androgen stimulation with DHT (Fig. 3). A plausible explanation for this discrepancy is that Xu et al. performed their localization studies in prostate cancer PC3 cells, whereas our study was performed in HEK-293 cells. Xu and colleagues reported that, similar to AR-FL/AR-V7 dimerization, AR-V7/AR-V7 dimerization was detected primarily in the nucleus. Our data point to the possibility that AR-FL forms dimers in the cytoplasm upon androgen stimulation and translocates into the nucleus, where AR-FL may interact with AR-V7 to form heterodimers. The differences in dimerization and luminescence kinetics further support our theory that for heterodimer formation, translocation of the AR-FL partner into the nucleus is necessary; therefore, these luminescence kinetics are slower.

AR-V7 resides constitutively in the nucleus [15, 27]. As stimulation with DHT induces the formation of the heterodimers of the AR-FL and AR-V7 proteins, we further assessed the effect of androgen stimulation on the subcellular localization of AR-FL and AR-V7 in AR protein-null HEK-293 cells by IF staining. Consistent with the findings of previous reports, AR-V7 was found exclusively in the nucleus, whereas AR-FL was localized predominantly in the cytoplasm under androgen-deprived conditions. However, as early as 15 min after androgen stimulation, AR-FL homodimers were found mainly in the nucleus (Fig. 3). Similarly, when measuring the luminescence of AR-FL/AR-V7, luminescent signals needed approximately 15 min to reach their maximum after the substrate was added (Fig. 4). We can hypothesize that the AR-FL/AR-V7 interaction occurs in the nucleus and not the cytoplasm. Nevertheless, we could not confirm this result by bioluminescent imaging due to the technical limitations of our approach.

Cao et al. reported that AR-V7 can cooccupy the promoter of the PSA gene with AR-FL [29]. Our theory that AR-FL and AR-V7 interact in the nucleus upon androgen stimulation is based on the following possibilities: 1. AR-FL and AR-V7 dimers may reside together at the androgen response elements (AREs), and their close proximity produces the detected NanoBiT luminescent signals or 2. Once in the nucleus, AR-FL and AR-V7 form heterodimers to modulate gene transcription.

To better characterize the time course of the AR-FL/AR-FL homodimers and AR-FL/AR-V7 heterodimers, we measured luminescence after androgen stimulation at 3 min intervals.

As expected, the AR-FL/AR-FL homodimer showed a luminescence signal after 3 min that continuously increased until 24 min after androgen stimulation and then remained stable for the following measured time points. Because of the early detection of the luminescence signal, we suggest that AR-FL/AR-FL homodimerization starts in the cytoplasm and that the homodimers are transported into the nucleus. This finding is in accordance with the model presented by Feldman and Feldman [9]. However, others suggest that homodimerization of AR-FL/AR-FL starts in the nucleus [2]. A possible explanation for this discrepancy is that AR-FL/AR-FL homodimerization can start in the cytoplasm, but enrichment of the homodimers is observed in the nucleus. However, AR-FL/AR-V7 heterodimer formation may occur differently. Luminescence, a marker for dimerization increased more slowly and linearly until 15 min and then remained at a constant level. We suggest that within 15 min after stimulation, AR-FL translocates into the nucleus, where it encounters AR-V7 homodimers to form AR-FL/AR-V7 heterodimers. The luminescence levels of AR-FL/AR-V7 are lower than those of AR-FL/AR-FL. This finding is in accordance with reduced fluorescence levels for AR-FL/AR-V7 constructs compared with those of the AR-FL/AR-FL constructs [4].

However, while we must consider that we and others have applied in vitro model systems, AR-V7 in the clinical setting is a dynamic marker that can change according to treatment conditions [37] and can also heterodimerize with other AR-Vs [38]. Interestingly, our research group observed general cytoplasmic and granular cytoplasmic staining patterns for AR-V7 via immunohistochemical staining on a tissue microarray with 410 primary PCa specimens, i.e., patients were not yet treated with ADT. However, AR-V7 nuclear staining occurred in only 25 patients (6.2%). AR-V7 granular staining was unexpectedly associated with longer relapse-free survival (RFS), whereas staining of the cytoplasm was associated with shorter RFS. More importantly, the granular staining pattern was similar to that of GOLGB1 (synonymous: giantin), a major protein of the Golgi apparatus. The coinciding staining pattern suggested that AR-V7 is localized in the Golgi apparatus [39]. When looking carefully at the AR-V7 IF stained images presented in this work, a granular fluorescent pattern could be distinguished around the nucleus, but due to the close proximity of the Golgi apparatus to the nucleus, determining the exact location was difficult. Considering the longer RFS associated with granular staining, we suggest that AR-V7 is not functionally active in these patients and may play a role in the protein degradation process in the Golgi apparatus. Li et al. described the proteasomal degradation of AR-V7 in prostate cancer cells controlled by protein phosphatase 1 [40].

There are different possibilities for the constitutive expression of AR-V7 in the nucleus. First, amino acids or their changes in the cryptic exon CE3 could be responsible for AR-V7 expression and localization. Chan et al. showed that K629A and R631A mutations in CE3 shifted AR-V7 expression from predominantly nuclear to a mixed nuclear/cytoplasmic pattern [27]. Nuclear import of AR-V7 is not mediated by the microtubule pathway but possibly by the importin α/β machinery [41]. Furthermore, Src family kinases have been identified as potential regulators of AR-V7 expression and AR-V7 localization [42]. Overall, the regulation of AR-V7 still appears to be a complex process.

The AR-V7 transcript has been detected in many different cancers and normal cell lines and in normal tissues, such as the liver, spleen, testis, skeletal muscle, small intestine, adipose tissue, and cervix [43]. AR-V7 may regulate wound repair via tenascin c [44]. Furthermore, Hu et al. and Cai et al. showed via gene set enrichment analysis that AR-V7 is involved in the activation of androgen-responsive, oncogenic (MYC and MYB), cell-cycle progression (E2F), and cancer-progression-associated genes [28, 45]. Several transcriptional targets uniquely activated by AR-V7 (e.g., ZNF32, FZD6, HDAC3, PHF21B, and SKP2) have been identified [28]. Taken together, these findings indicate that AR-V7 can induce a specific transcriptional program of genes that function mostly as oncogenes, which exacerbates PCa. Therefore, AR-V7 may still receive special consideration as a future therapeutic target in CRPC beyond AR.

There are limitations in our study. We did not study a PCa cell line that would be somewhat representative of a PCa. We performed our experiments in human embryonic kidney (HEK) 293 cells, where we could study the interactions and localization of AR-FL and AR-V7 without disturbance by an intrinsic PCa-related activation of AR signaling. Our signal strength determined via luminescence microscopy was not sufficient to localize our constructs in time or at the subcellular level. Therefore, to overcome this technical limitation, future experiments in an Olympus LV200 bioluminescence imager, which has been described to perform bioluminescence imaging with sufficient resolution to clearly detect and localize luminescence signals [46], would be necessary.

## Conclusions

AR-FL is initially located as a monomer in the cytoplasm before DHT treatment and possibly as an AR-FL/AR-FL homodimer shortly after DHT treatment. AR-FL/AR-FL homodimers translocated into the nucleus within 15 min after DHT treatment. AR-V7/AR-V7 homodimers were constitutively located in the nucleus, and neither DHT nor enzalutamide affected the localization of AR-V7/AR-V7 or its status as a dimer. AR-FL/AR-V7 heterodimers form only after DHT stimulation. Our data indicate that AR-V7/AR-FL heterodimers form in the nucleus after AR-FL homodimers are translocated to the nucleus.

### Supplementary Information

**Supplementary Material 1. **

## Data Availability

All the data are available in the manuscript and the Supplementary Materials. The detailed datasets used and analyzed during the present study are available from the corresponding author upon reasonable request.

## Abbreviations

ADT: Androgen deprivation therapy
AR: Androgen receptor
AR-V7: Androgen receptor splice variant 7
CRPC: Castration resistant prostate cancer
DHT: Dihydrotestosterone
HEK: Human embryonic kidney cells
LgBiT: LargeBiT
NLS: Nuclear localization signal
PCa: Prostate cancer
SmBiT: SmallBiT
