# Supplementary material for: NanoLuc Binary Technology as a methodological approach: an important new tool for studying the localization of androgen receptor and androgen receptor splice variant V7 homo and heterodimers
Source: BMC Cancer. 2024 Mar 19;24:346. doi: 10.1186/s12885-024-12110-2 (PMC10949640; doi:10.1186/s12885-024-12110-2)
Supplement: Supplementary file 1 — Supplementary Material 1. [file 12885_2024_12110_MOESM1_ESM.docx]

Suppl. Data

NanoLuc Binary Technology as a methodological approach: An important new tool for studying the localization of androgen receptor and androgen receptor splice variant V7 homo- and heterodimers

Juan Guzman ^1,2^, Katrin Weigelt ^1,2^, Angela Neumann ^1,2^, Philipp Tripal ^3^, Benjamin Schmid ^3^, Zoltán Winter ^3^, Ralph Palmisano ^3^, Zoran Culig ^4^, Marcus V. Cronauer ^5^, Paul Muschler ^6^, Bernd Wullich ^1,2^, Helge Taubert ^1,2,*,†^ and Sven Wach ^1,2,†^

**Suppl. Table S1. List of plasmids used and cloned in this study**

| **Name** | **Gene** | **Resistance** |
| --- | --- | --- |
| pEGFP-AR-WT | Androgen receptor | Kanamycin |
| pEGFP-AR-V7 | Androgen receptor splice variant-7 | Kanamycin |
| pBiT1.1-N[TK/LgBiT] | Cloning NanoBiT Expression Vector | Ampicillin |
| pBiT1.1-C[TK/LgBiT] | Cloning NanoBiT Expression Vector | Ampicillin |
| pBiT2.1-N[TK/SmBiT] | Cloning NanoBiT Expression Vector | Ampicillin |
| pBiT2.1-C[TK/SmBiT] | Cloning NanoBiT Expression Vector | Ampicillin |
| NanoBiT Negative Control | Negative control for Nano-Glo Live Cell Assay System | Kanamycin |
| LgBiT-PRKAR2A Control | Positive control for Nano-Glo Live Cell Assay System | Kanamycin |
| SmBiT-PRKACA Control | Positive control for Nano-Glo Live Cell Assay System | Kanamycin |
| pBiT1.1-N[TK/LgBiT] + AR | NanoBiT construct with the OPF of the Androgen receptor | Ampicillin |
| pBiT1.1-C[TK/LgBiT] +AR-V7 | NanoBiT construct with the OPF of the Androgen receptor splice variant-7 | Ampicillin |
| pBiT2.1-N[TK/SmBiT] +AR | NanoBiT construct with the OPF of the Androgen receptor | Ampicillin |
| pBiT2.1-C[TK/SmBiT] + AR-V7 | NanoBiT® construct with the OPF of the Androgen receptor splice variant-7 | Ampicillin |

**Suppl. Table S2. Workflow of immunofluorescence detection**

| **Timeline** | **HEK-293** |
| --- | --- |
| Day 0 Coating with Poly-L-Lysine | 500 µL |
| Day 0 Seeding | 200,000 cells |
| Day 1 Transfection | 2 µg DNA |
| Day 2 Stimulation with 1 nM DHT | 2.2 µL |
| Day 2 Fixation with 4 % PFA | 500 µL |
| Day 2 Permeabilization with 0.2% Triton X-100 | 500 µL |
| Day 2 Blocking with 1% BSA + 0.1% Triton X-100 | 500 µL |
| Day 2 Incubation with NanoLuc Luciferase Antibody (1:500) | 1000 µL |
| Day 3 Incubation with the Alexa Fluor 488 antibody (1:1000) | 1000 µL |
| Day 3 Preservation with Mounting Medium containing DAPI | 100 µL |


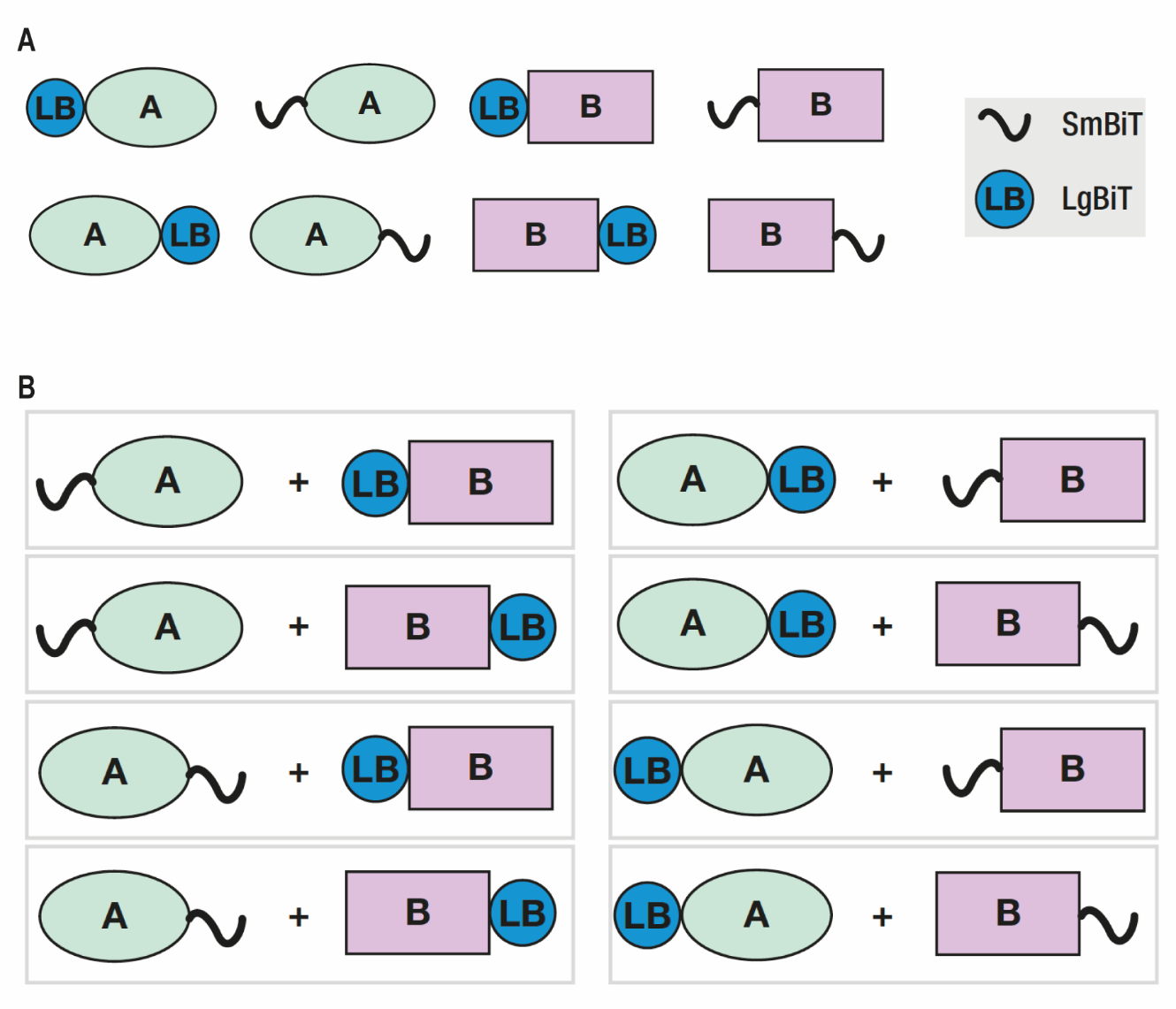


**Suppl. Figure S1. Principle of the NanoBiT Protein:Protein Interaction system**

Each protein of interest is fused at either N or C terminus with LgBiT and SmBiT (A) This creates up to eight different expression constructs. Here the androgen receptor and the androgen receptor splice variant AR-V7 are the proteins of interest. (The figure is copied with permission from the Promega Corporation: NanoBiT Protein:Protein Interaction System Technical Manual).





**Suppl. Figure S2. Expression of fusion constructs**

Lane1: DuCaP constitutively express AR-FL and AR-V7. Plasmid combinations AR-FL-SmBit/AR-FL-LgBit (Ho3; lane 2), AR-V7-SmBit/AR-V7-LgBit (Ho5; lane 3) and AR-FL-SmBit/AR-V7-LgBit (He3; lane 4) were expressed in HEK293 cells. The figure is cropped. It consists of the same blot incubated with the AR antibody (D6F11, 1:1000, Cell Signaling; upper part) and the GPDH antibody (14C10, 1:1000, Cell Signaling; lower part).





**Suppl. Fig.S3. Original exposures used for composing Suppl. Fig. S2**


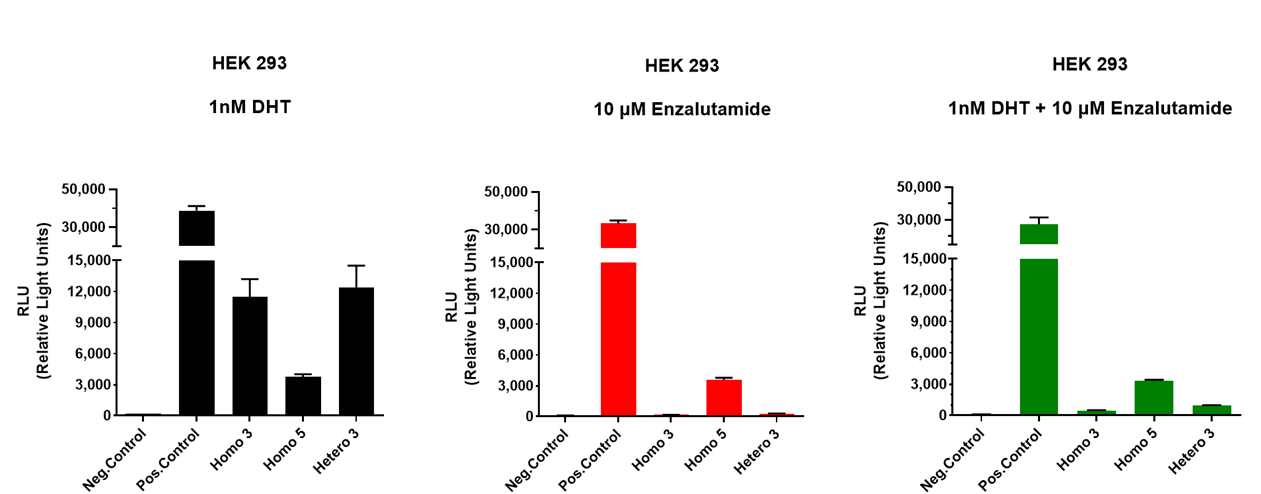


**Suppl. Figure S4.** Nano-Glo Live Cell assay

HEK-293 cells were transfected with combinations of homo- and heterodimers encoding the androgen receptor full length (AR-FL) and androgen receptor splice variant 7 (AR-V7) proteins. Luminescence measurements in cells after treatment with 1 nM DHT, 10 µM enzalutamide or combination of DHT/enzalutamide was measured. The data represent the mean ± SEM (n=3).
